# Bone morphogenetic protein inhibitors and mitochondria targeting agents synergistically induce apoptosis-inducing factor (AIF) caspase-independent cell death in lung cancer cells

**DOI:** 10.1186/s12964-022-00905-4

**Published:** 2022-06-27

**Authors:** Arindam Mondal, Jacques Roberge, John Gilleran, Youyi Peng, Dongxuan Jia, Moumen Akel, Yash Patel, Harrison Zoltowski, Anupama Doraiswamy, John Langenfeld

**Affiliations:** 1 Department of Surgery, Rutgers Robert Wood Johnson Medical School, Rutgers, The State University of New Jersey, 1 Robert Wood Johnson Place, New Brunswick, NJ 08903 USA; 2 Molecular Design and Synthesis, RUBRIC, Office for Research, Rutgers Translational Science, Rutgers University, Piscataway, NJ 08854 USA; 3 Biomedical Informatics Shared Resources, Rutgers Cancer Institute of New Jersey, New Brunswick, NJ 08903 USA; 4 Cancer Institute of New Jersey, New Brunswick, NJ USA; 5 Rutgers University, Piscataway, NJ 08854 USA

**Keywords:** BMP, AMPK, Cell death, AIF, Caspase-independent

## Abstract

**Background:**

Bone morphogenetic proteins (BMP) are evolutionarily conserved morphogens that are reactivated in lung carcinomas. In lung cancer cells, BMP signaling suppresses AMP activated kinase (AMPK) by inhibiting LKB1. AMPK is activated by mitochondrial stress that inhibits ATP production, which is enhanced 100-fold when phosphorylated by LKB1. Activated AMPK can promote survival of cancer cells but its “hyperactivation” induces cell death. The studies here reveal novel cell death mechanisms induced by BMP inhibitors, together with agents targeting the mitochondria, which involves the “hyperactivation” of AMPK.

**Methods:**

This study examines the synergistic effects of two BMP inhibitors together with mitochondrial targeting agents phenformin and Ym155, on cell death of lung cancer cells expressing LKB1 (H1299), LKB1 null (A549), and A549 cells transfected with LKB1 (A549-LKB1). Cell death mechanisms evaluated were the activation of caspases and the nuclear localization of apoptosis inducing factor (AIF). A769662 was used to allosterically activate AMPK. Knockdown of BMPR2 and LKB1 using siRNA was used to examine their effects on nuclear localization of AMPK. Validation studies were performed on five passage zero primary NSCLC.

**Results:**

Both BMP inhibitors synergistically suppressed growth when combined with Ym155 or phenformin in cells expressing LKB1. The combination of BMP inhibitors with mitochondrial targeting agents enhanced the activation of AMPK in lung cancer cells expressing LKB1. Allosteric activation of AMPK with A769662 induced cell death in both H1299 and A549 cells. Cell death induced by the combination of BMP inhibitors and mitochondrial-targeting agents did not activate caspases. The combination of drugs induced nuclear localization of AIF in cells expressing LKB1, which was attenuated by knockdown of LKB1. Knockdown of BMPR2 together with Ym155 increased nuclear localization of AIF. Combination therapy also enhanced cell death and AIF nuclear localization in primary NSCLC.

**Conclusions:**

These studies demonstrate that inhibition of BMP signaling together with mitochondrial targeting agents induce AIF caspase-independent cell death, which involves the “hyperactivation” of AMPK. AIF caspase-independent cell death is an evolutionarily conserved cell death pathway that is infrequently studied in cancer. These studies provide novel insight into mechanisms inducing AIF caspase-independent cell death in cancer cells using BMP inhibitors.

**Video Abstract**

**Supplementary Information:**

The online version contains supplementary material available at 10.1186/s12964-022-00905-4.

## Background

Bone morphogenetic protein (BMP) signaling is a conserved regulator of cell-fate decisions of stem cells throughout embryonic development. BMP signaling is aberrantly expressed in age-related diseases including Alzheimer’s disease [1–4], obesity [5], non-small cell lung carcinoma (NSCLC) and other carcinomas [6, 7]. BMP2 ligand is highly overexpressed in NSLC in comparison to normal lung and benign tumors [8]. BMP ligands signal through receptor serine/threonine kinases [9]. BMP ligands bind to the BMP type 1 receptors (BMPR1) (alk2, alk3, or alk6) promoting phosphorylation by the constitutively active BMP type 2 receptors (BMPR2) (BMPR2, ActR-IIA, ActRIB) [9]. The BMP complex phosphorylates Smad-1/5, which induces transcription of the inhibitor of differentiation proteins (Id1, Id2, and Id3) [10–12] and other transcriptional events. BMP signaling also promotes survival of cancer cells by regulating BMPR2 independent of Smad-1/5. BMPR2 mediated signaling leads to the activation of PI3K/Akt [13–16] and increases the expression of the anti-apoptotic proteins, X chromosome-linked inhibitor of apoptosis protein (XIAP) and transforming growth factor beta (TGFβ) activated kinase 1 (TAK1) [17–19].

BMP signaling has recently been reported to regulate catabolic metabolic signaling in NSCLC cell lines. BMP signaling suppresses AMP activated kinase (AMPK) in NSCLC lung cancer cell lines, which is conserved in *C elegans* [20]. Inhibition of BMP receptor signaling with small molecules or loss of function transgenes induces AMPK activation in lung cancer cells and *C elegans* [20]. In lung cancer cell lines, BMP signaling was found to inhibit AMPK by suppressing LKB1 [20]. Activated AMPK induces a negative feedback regulation of BMP signaling in lung cancer cell lines and *C elegans* [20]. AMPK is activated by a decrease in ATP levels and cellular stress. To conserve ATP, AMPK inhibits anabolic signaling which includes the inhibition of fatty acid, cholesterol, glycogen, and protein synthesis [21]. Although AMPK can promote survival of cancer cells during cellular stress, hyperactivation of AMPK is reported to promote cell death [22]. The role of AMPK activation regulating cancer cell death following the inhibition of BMP signaling is not known.

We recently reported that cancer therapeutics, which activate AMPK, cause a decrease in BMP signaling. Ym155 binds to the mitochondrial DNA, leading to a decrease in oxidative phosphorylation and decrease in tricarboxylic acid (TCA) cycle metabolites. Ym155 causes potent activation of AMPK and decreases BMP signaling at 10 nM concentrations in lung cancer cell lines [23]. The mitochondrial complex II inhibitor, Phenformin [24], also activates AMPK and suppresses BMP signaling in lung cancer cell lines. Suppression of mitochondrial function decreases the synthesis of ATP leading to an increase in AMP. AMP binding to the *γ* regulatory unit causes allosteric activation of AMPK by approximately tenfold [25]. To be fully activated, AMPK also needs to be phosphorylated at Thr172 by Liver Kinase B1 (LKB1), which can cause a 100-fold activation [25]. These studies raise the question whether “hyperactivation” of AMPK can be induced when combining a mitochondrial inhibitor together with a BMP inhibitor and what impact this would have on the survival of cancer cells.

In the present paper, we examined whether AMPK activation in lung cancer cells regulates cell survival. We also examined whether combining the mitochondrial inhibitors, Ym155 and phenformin, with BMP receptor inhibitors enhances AMPK activation and affects cell death signaling. We find that AMPK promotes cell death in lung cancer cell lines. BMP inhibition with small molecule receptor antagonists together with Ym155 or Phenformin synergistically induced cell death and enhanced AMPK activation in lung cancer cells expressing LKB1. Activated AMPK induced by BMP inhibition together with Ym155 or phenformin caused AIF to localize to the nucleus, which occurs independent of caspase activation. These studies suggest a novel means to induce AIF caspase-independent cell death in lung cancer cells, which involves “hyperactivation” of AMPK induced by BMP inhibition together with a mitochondrial inhibitor.

## Methods

### Cell culture and reagents

The A549 and H1299 lung cancer cells were cultured in Dulbecco’s modified Eagle’s medium (DMEM, Sigma Aldrich, Saint Louis, MO, USA) with 5% fetal bovine serum (R&D Systems). JL5, DMH2, and JL189 were synthesized by John Gilleran, Anastasia Tsymbal, and Jacques Roberge, Rutgers School of Pharmacy. Ym155 was purchased from Selleckchem. Z-VAD-FMK was purchased from R&D Systems. SB-505124 and necrostatin-1 were purchased from Sigma Aldrich (Saint Louis, MO, USA).

### Western blot analysis

Western blot analysis was performed as previously reported [26]. In brief, total cellular protein concentration was determined using the BCA method then separated by SDS-PAGE and transferred to nitrocellulose (Schleicher and Schuell, Keene, NH). The primary antibodies used were rabbit monoclonal anti-XIAP, rabbit monoclonal anti-Smac/DIABLO, rabbit monoclonal anti-cytochrome c, rabbit monoclonal anti-c-IAP1, rabbit monoclonal anti-activated caspase-3, mouse monoclonal anti-PARP (BD Pharmingen, USA), rabbit monoclonal anti-AIF, rabbit monoclonal anti-pSmad1/5, (Cell signaling Technology, MA, USA), rabbit monoclonal anti-Id1 (Calbioreagents, San Mateo, CA), rabbit polyclonal anti-Smad 1/5 (Upstate Biotechnology, NY, USA), rabbit polyclonal survivin (Novus Biologicals, CO, USA), rabbit anti-actin, an affinity isolated antigen specific antibody (Sigma, Saint Louis, MO), rabbit polyclonal anti-GAPDH (Sigma) and mouse monoclonal anti-Spectrin (EMD Millipore, CA, USA).

Blots suggesting regulation of loading controls actin or GAPDH by Ym155 and/or JL5 were then probed with spectrin, which is also a cytoskeletal protein.

### Cell viability

Cells were plated in duplicate into 6-well plates and treated the next day for the designated time period. Cell counts were determined using the automated cell counter Vi-CELL cell analyzer (Beckman Coulter). Approximately 500 cells per sample were analyzed and trypan blue dye exclusion determined the number of dead cells. The experiment was replicated three times in our laboratory.

### Combined drug effects

The median-effect principle of Chou and Talalay was used to evaluate synergy between JL5 and Ym155 [27]. The combination index (CI) values were calculated using Compusyn software to determine mode of interaction with CI < 1.0 indicating synergism, 1.0 additive and > 1.0 antagonistic response.

### Transient knockdown

Validated select siRNA was used to knockdown BMPR2 and LKB1 (Life Technologies). The ID numbers for the siRNA are: BMPR2 (s2044 and s2045), LKB1 (s13579). Silencer Select negative control siRNA (4390843) was used to evaluate selectivity. Transfections of the siRNA were performed in duplicate using Lipofectamine® RNAiMAX Reagent (Invitrogen, Carlsbad, CA, USA) according to manufacturer’s protocol. Cells were transfected with 6 nM BMPR2 or 6 nM of siRNA control. Cells were also transfected with 12 nM siLKB1 and 12 nM of siRNA control. The experiment was replicated three times in our laboratory.

### Cytosol extraction

Cytosolic protein extraction was performed using Mitochondria/Cytosol fractionation kit as per manufacturer’s instructions (Enzo Life Sciences, NY, USA). 750,000 cells/ well were grown overnight and then treated for designated period. Cell pellets were resuspended in 100 µl of ice-cold Cytosol Extraction Buffer Mix containing dithiothreitol (DTT) and Protease Inhibitors. After a 10-min incubation on ice, cells were homogenized. The homogenates were collected to a fresh 1.5 ml tube and centrifuged at 700×*g* for 10 min at 4 °C. The supernatant was collected as the cytosolic fraction and used for further experiments. The supernatant was collected and centrifuged at 10,000×*g* for 30 min at 4 °C. The supernatant was collected as the cytosolic fraction and used for further experiments.

### TUNEL assay

DNA double strand breaks (DSB) after treatment were analyzed by using FlowTACS In Situ TUNEL-based apoptosis detection kit (Trevigen) according to the manufacturer’s protocol. Cells were treated in duplicate. After treatment, cells were trypsinized and the cell pellet was fixed with 4% formaldehyde and permeabilized with cytonin for 30 min. Cells were washed with labeling buffer and resuspended in reaction mix for 1 h (h), then stained with strep-fluorescein solution and analyzed using flow cytometry (LSRII, BD Biosciences). The experiment was replicated three times in our laboratory.

### Immunofluorescence staining

Cells were seeded for 24 h onto microscope cover glasses in a 6-well plate and then treated. Cells were fixed with 4% formaldehyde and permeabilized with 0.5% triton-X. Cells were blocked with CAS-block for 1 h. Cells were stained with anti-BMPR2 antibody that recognizes an extracellular epitope (Sigma-Aldrich) or AIF for 1 h at room temperature. To assess for BMPR2 on the plasma membrane,the cells were fixed but not permeabilized prior to staining. Cells were then washed with phosphate buffered saline (PBS) and stained with Alexa Flour 488 conjugated secondary antibody for 1 h at room temperature. After washing with PBS, the nuclei were counterstained with 4′,6-diamidino-2-phenylindole (DAPI) (Sigma-Aldrich) for 10 min. Fluorescent images were captured using a Nikon eclipse TE300 inverted epifluorescent microscope and a Cool Snap black and white digital camera. IP Lab imaging software was used to assign pseudo-color to each channel. All the immunofluorescence experiments, except primary tumor cells, were replicated three times in our laboratory. The AIF immunofluorescence study using primary tumor cell lines was performed once for each of the 5 tumors.

### Primary lung carcinomas

Primary tumor tissues were obtained within 30 min of surgery from The Cancer Institute of New Jersey (CINJ) as approved by the Research Ethics and Institutional Review Board (Protocol Number: 001608). Consecutive non-small cell lung carcinomas were used. The tissues were washed with PBS and then cut into small pieces. Human tissue dissociation kit (Miltenyl Biotec) was used to dissociate the tumor tissues according to the manufacturer’s protocol. Dissociated cells were grown in Dulbecco’s Modified Eagle Medium (DMEM) media supplemented with 20% Fetal Bovine Serum (FBS) and 1% antibiotic–antimycotic for approximately 10 days without splitting. At 70% confluence, cells were trypsinized and plated for all the experiments at the same time.

### Statistical analysis

In lung cancer studies, paired student *t*-test assuming unequal variances was used to compare means. The mean of control was compared with the mean of each treated group. Differences with *p* values < 0.05 were considered statistically significant. The following signified *p < 0.05, **p ≤ 0.01, ***p ≤ 0.001, ****p ≤ 0.0001.

## Results

### Mitochondrial inhibitors induce more cell death in lung cancers expressing LKB1

**Fig. 1:**
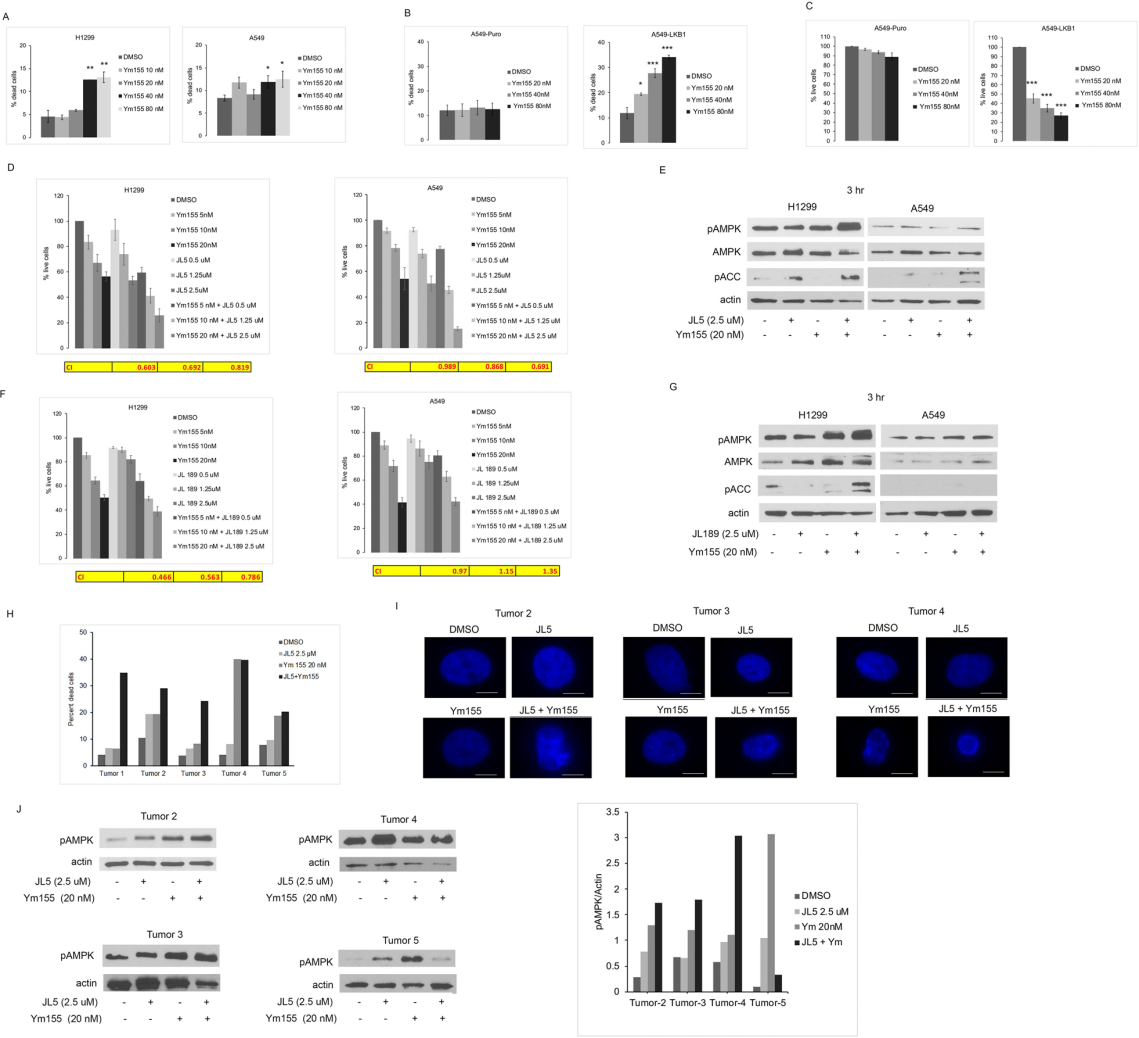
Mitochondrial inhibitor Ym155 and BMP inhibitors enhance AMPK activation and induce more cell death in LKB1 competent cells. **A** Mean percent cell death of 4 experiments of H1299 and A549 cells treated with Ym155 for 24 h. Mean percent death and percent live cells of 4 experiments of A549-LKB1 and A549 wild-type cells treated with Ym155 (**B**, **C**). **D**, **F** Mean percent live cells of 4 experiments of H1299 and A549 cells treated with BMP inhibitors JL5, JL189 or Ym155 alone and in combination for 48 h. Yellow table represents the combination index (CI) of cells treated with Ym155 plus BMP inhibitor at each concentration. Concentration of drugs used increase from left to right. **E**, **G** Representative immunoblots of A549 and H1299 cells treated with BMP inhibitors JL5, JL189, or Ym155 alone and in combination for 24 h. **H** Cell counts of primary lung cancer samples treated with JL5 and Ym155 alone and in combination for 48 h. **I** Primary lung cancer samples were treated for 36 h and nuclei stained with DAPI. Scale bar is equal to 10 μm. (J) Western blot analysis of primary tumors

Ym155 activates AMPK in H1299 cells, which harbor wild-type LKB1. In A549 cells, which lack LKB1, Ym155 does not fully activate AMPK. Ym155 induced more cell death in H1299 cells compared to A549 cells (Fig. 1A). A549 cells, in which LKB1 transgene is stably transduced, Ym155 caused significantly more cell death and decrease in the number of live compared to A549 wild-type cells after 24 h (Fig. 1B, C). These studies suggest that activation of AMPK may have a role in promoting cell death in lung cancer cells.

### BMP inhibitors and Ym155 synergistically suppress growth and induce cell death of lung cancer cells

JL5 is a potent inhibitor of the BMP type I receptors with some inhibition of BMPR2 [6]. JL5 together with Ym155 induced more cell death and caused greater growth inhibition than either compound alone (Fig. 1D, Additional file 1: Fig. S1). A Combination Index (CI) of < 1.0 indicates synergy, = 1 indicates additive effect, and > 1.0 indicates an inhibitory effect. The CI in both H1299 and A549 cells demonstrated synergy when treated with JL5 combined with Ym155 but was more pronounced in H1299 cells. Western blot analysis showed the combination of JL5 and Ym155 demonstrated higher levels of AMPK phosphorylation at Thr172 [28] in H1299 cells but not A549 cells (Fig. 1E). Activated AMPK phosphorylates acetyl-CoA carboxylase (ACC) at Ser79 leading to its activation. JL5 when combined with Ym155 increased pACC (Ser79) levels higher than either compound alone in both H1299 and A5499 (Fig. 1E). These studies suggest that JL5 together with Ym155 enhance full activation of AMPK in H1299 but also increases allosteric activation of AMPK in A549 cells.

JL189 is small molecule that inhibits BMPR2 more than BMP type 1 receptors (Additional file 2: Table S1). JL189 in combination with Ym155 synergistically inhibited growth and enhanced cell death of H1299 cells but not A549 cells (Fig. 1F, Additional file 1: Fig. S1). The combination of JL189 and Ym155 also enhanced pAMPK (Thr172) and pACC (Ser79) levels in H1299 cells but not in A549 cells (Fig. 1G). Together, these studies show that pharmaceutical inhibition of BMP signaling together with the mitochondrial inhibitor Ym155 enhances AMPK activation and synergistically inhibits growth and induces cell death predominantly in LKB1 competent lung cancer cells.

### Synergy of Ym155 and JL5 in primary NSCLC

Primary NSCLC cells were obtained directly from 5 surgically resected lung tumors. Tumors 1, 2, 3 and 5 were adenocarcinomas and tumor 4 was a squamous carcinoma. Tumors were immediately gently digested and plated for cell culture. After approximately 10 days the cells were treated with JL5 and Ym155 alone and in combination, for 48 h. The combination of JL5 and Ym155 caused an increase in cell death compared to DMSO control in 5 of 5 tumors (Fig. 1H). In 3 of 5 tumors, the combination of JL5 and Ym155 induced cell death that was greater than either compound alone (Fig. 1H). Morphologically, the nuclei were significantly smaller and demonstrated chromatin condensation when treated with both JL5 and Ym155 in comparison to either compound alone (Fig. 1I). Western blot analysis demonstrated enhanced expression of pAMPK with the combination of JL5 and Ym155 compared to each alone in tumors 2, 3, and 4 but not in tumor 5 (Fig. 1J).

### Phenformin together with BMP inhibitors synergistically suppress growth and induce cell death

**Fig. 2:**
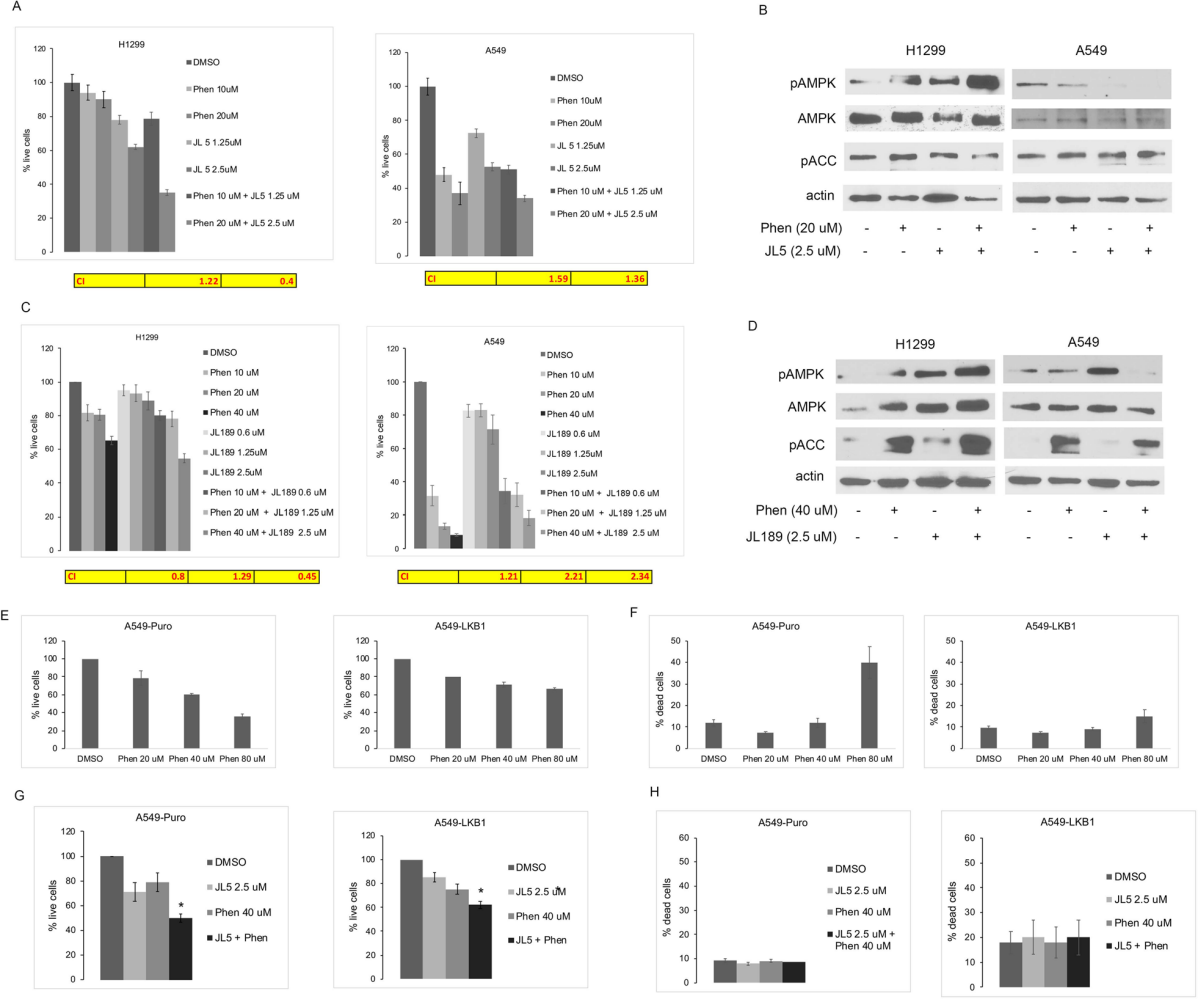
Synergistic growth suppression and enhanced AMPK activation with BMP inhibition when combined with phenformin in LKB1 competent cells. **A**, **C** Mean percent live cells of 4 experiments of H1299 and A549 cells treated with BMP inhibitors JL5, JL189, or phenformin alone and in combination for 48 h. Yellow table represents the combination index (CI) of cells treated with phenformin plus BMP inhibitor at each concentration. Concentration of drugs used increase from left to right. **B**, **D** Representative immunoblot of A549 and H1299 cells treated with BMP inhibitors JL5, JL189, or phenformin alone and in combination for 24 h. **E**, **F** Mean live and dead cells of A549-Puro and A549-LKB1 cells treated with phenformin for 48 h, n = 4. **G**, **H** Mean live and dead cells of A549-Puro and A549-LKB1 cells treated with phenformin and JL5 for 48 h, n = 4

Phenformin inhibits complex I of the electron transport chain and activates AMPK [30]. Phenformin in combination with JL5 or JL189 synergistically suppressed growth and induced cell death of H1299 cells but not A549 cells (Fig. 2A, C, Additional file 3: Fig. S2A-B). The combination of phenformin with JL5 or JL189 increased pAMPK (Thr172) levels in comparison to each compound alone in H1299 cells but not A549 cells (Fig. 2B, D). These studies again demonstrate that pharmaceutical inhibition of BMP signaling in combination with a mitochondrial inhibitor enhances AMPK activation and synergistically suppresses growth and induces cell death of LKB1 competent lung cancer cells.

A549-Puro cells treated with were less responsive to phenformin compared to wild-type A549 cells. A549-Puro and A549-LKB1 showed no increase in cell death up to 40 μM of phenformin (Fig. 2E), while 73% of A549 wild-type cells were dead with 20 μM (Additional file 3: Fig. S2). The combination of JL5 with phenformin caused more growth suppression than either compound alone in both A549-Puro and A549-LKB1 cells (Fig. 2G). However, the combination of JL5 and phenformin had no effect on cell death (Fig. 2H). These studies indicate that A549-Puro and A549-LKB1 are more resistant to cell death induced by phenformin than wild-type A549 cells Although A549-Puro cell do not behave like wild-type cells, these studies suggest that JL5 combined with phenformin suppresses growth by mechanism other than the activation of AMPK. However, the studies with A549-Puro and A549-LKB1 did not clarify the effects of AMPK on cell death when using a BMP inhibitor with phenformin.

### AMPK induces cell death

**Fig. 3:**
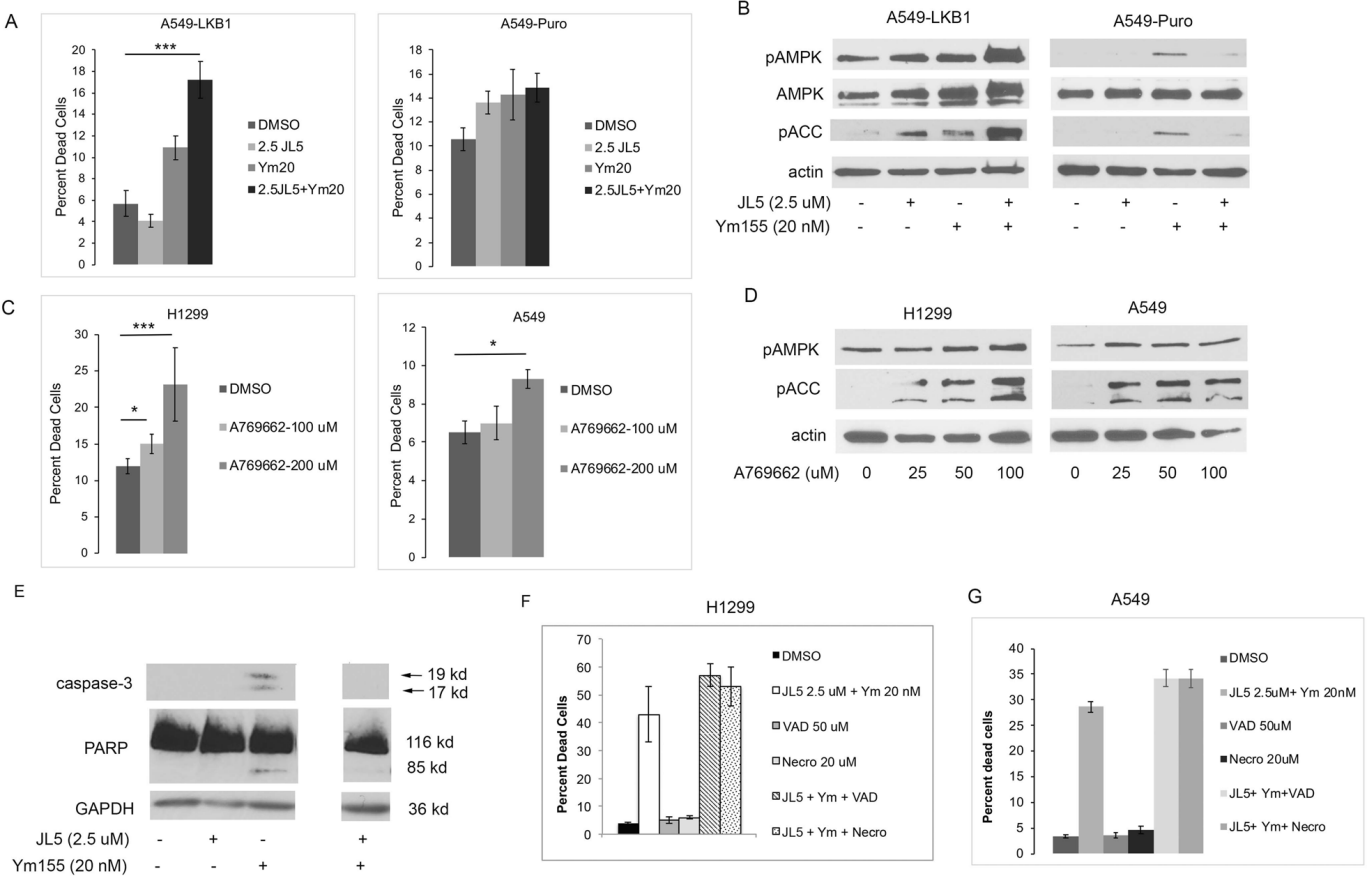
AMPK activation induces caspase-independent cell death. **A** Percent dead cells of A549 expressing LKB1 transgene (A549-LKB1) and A549 LKB1 null transfection control cells (A549-Puro) treated with JL5 and Ym155 alone and in combination for 48 h. Data represents mean of 6 studies. **B** Western blot analysis of A549-LKB1 A549-Puro cells treated for 24 h. **C** Percent death cells of H1299 cells treated with AMPK allosteric activator for 48 h. Data represents the mean of 4 studies. **D** Immunoblots of cells treated for 24 h. **E** Immuno blot of cells treated for 24 h. **F**, **G** Mean percentage of dead H1299 cells after treatment with JL5 and Ym155 in combination for 24 h with and without Z-VAD-FMK or necrostatin, n =  = 4

Since Ym155 induced more cell death in A549-LKB1 cells compared to A549-Puro cell, we further examined the role of AMPK inducing cell death. A549 reconstituted with LKB1 transgene and A549-Puro cells were treated with JL5 and Ym155 alone and in combination. The combination of JL5 with Ym155 significantly increased cell death compared to either compound alone in A549-LKB1 cells but not in wild-type A549 cells (Fig. 3A). JL5 and Ym155 in combination but not alone also increased pAMPK (Thr172) levels in A549-LKB1 cells but not in A549-Puro cells (Fig. 3B). The allosteric AMPK activator, A769662, significantly induced cell death and AMPK activation in H1299 cells and in A549 cells (Fig. 3C-D). These studies suggest that activated AMPK enhances cell death, which may be dependent on the level of activation and/or the type of cellular stress.

### Cell death is independent of caspase activation

Next, we examined the type of cell death induced by Ym155 and BMP inhibition. Ym155 alone induced apoptosis as demonstrated by the expression of activated caspase-3, with 17 kd and 19 kd fragments and cleavage of its downstream target, poly ADP ribose polymerase (PARP) (Fig. 3E). JL5 in combination with Ym155, we found no activation of caspase-3 or caspase-9 when examined at 1, 3, and 24 h (Fig. 3E and data not shown). The pan-caspase inhibitor Z-VAD-FMK did not affect cell death induced by JL5 and Ym155 in combination in either the H1299 or A549 cells (Fig. 3F-G). Necrostatin, which inhibits necrosis induced by receptor-associated adaptor kinase 1 (RIP1) [29], also had no effect on cell death induced by JL5 and Ym155 when used in combination (Fig. 3F, G). These studies suggest that Ym155 together with a BMP receptor inhibitor synergistically mediates cell death by mechanisms independent of the caspases or RIP1 induced necrosis.

### Ym155 together with JL5 increases cytosolic AIF and Smac/DIABLO

**Fig. 4:**
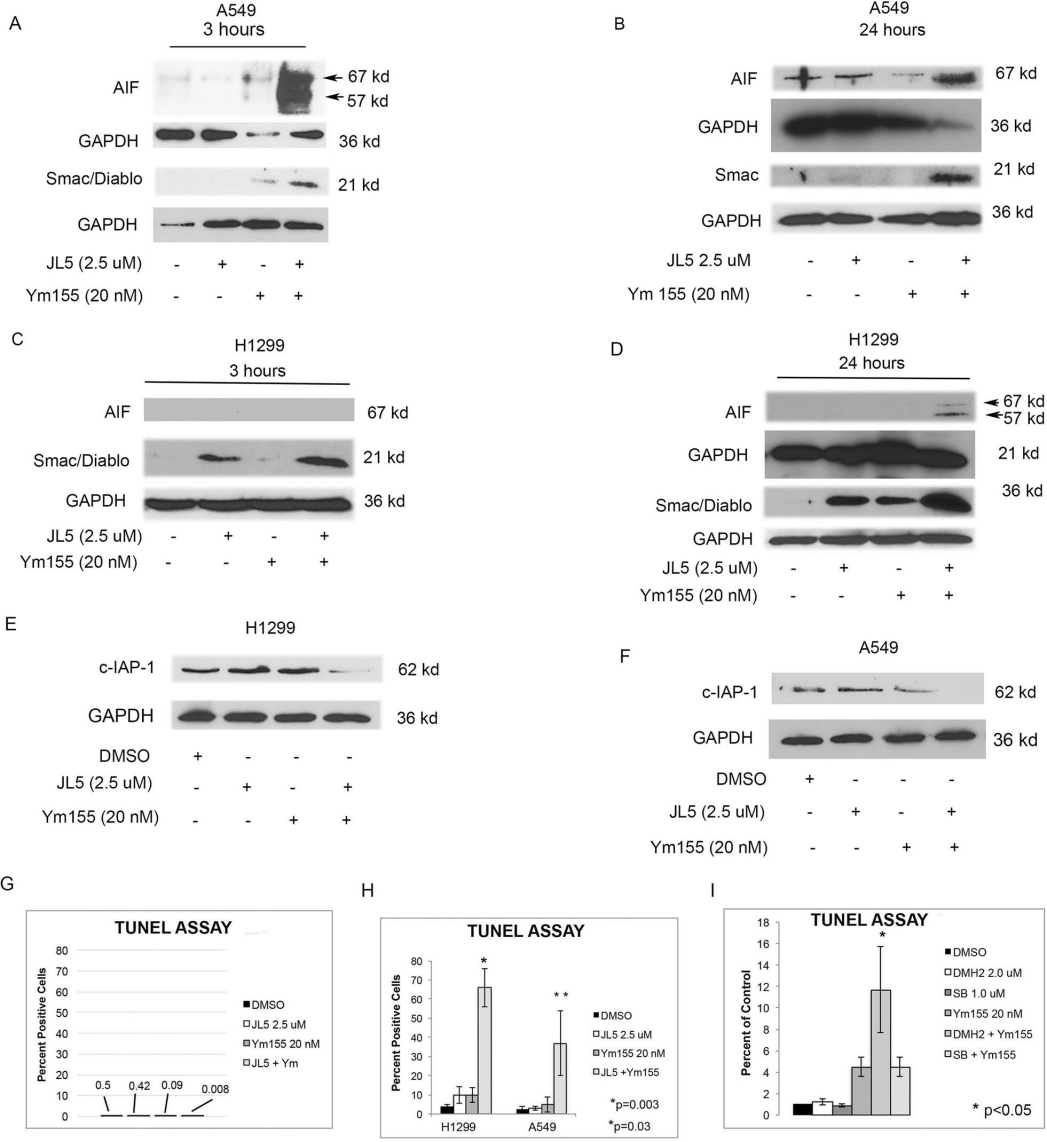
Ym155 in combination with JL5 increases cytosolic AIF. **A**–**D** Western blot analysis of cytosol of A549 and H1299 cells treated with JL5 and Ym155 alone and in combination for 3 or 24 h. Arrowheads demonstrate the 67 kd and 57 kd fragments of AIF, which only occur in cells treated with JL5 and Ym155 in combination. **E**–**F** Western blot analysis of cells treated for 24 h demonstrating a decrease expression of anti-apoptotic proteins when cells are treated with JL5 and Ym155 in combination. **G** TUNEL assay of H1299 cells were treated with JL5 and Ym155 alone and in combination for 3 h. **H** TUNEL assay of H1299 and A549 cells treated with JL5 and Ym155 alone and in combination for 24 h. **I** TUNEL assay of H1299 cells treated with DMH2, SB-505124 (SB), alone or in combination with Ym155 for 24 h. **G**, **H** Data represents the mean of 4 independent experiments

Since apoptosis is not induced with JL5 and Ym155 in combination, we explored whether cell death involved the mitochondrial release of AIF [31]. In cells undergoing AIF induced cell death, AIF is cleaved producing 67 kd and 57 kd fragments, which are then rapidly transported to the nucleus. Nuclear AIF induces DNA fragmentation and chromatin condensation, leading to cell death [31]. The combination of Ym155 and JL5 enhanced mitochondrial release of AIF and Smac/DIABLO into the cytosol as early as 3 h and persisted for at least 24 h (Fig. 4A–D). Cytosolic AIF was not observed when JL5 and Ym155 were used alone. Smac/DIABLO causes the inhibition and degradation of anti-apoptotic proteins cellular inhibitor of apoptosis1 (c-IAP-1). Consistent with an increase in cytosolic Smac/DIABLO, JL5 together with Ym155 caused a decrease in the expression of c-IAP-1 when used in combination but not when used alone (Fig. 4E–F).

### BMP inhibitors combined with YM155 cause DNA double stranded breaks

A hallmark of AIF induced cell death is the induction of DNA double stranded breaks (DSB) and its localization to the nucleus. The TUNEL assay was used to determine DNA-DSB. Three hours following treatment with JL5 or Ym155 alone or in combination, no DNA-DSB were found (Fig. 4G). After 24 h, very few cells treated with JL5 or Ym155 alone demonstrated DNA-DSB. When JL5 was used in combination with Ym155, approximately 40% and 65% of cells demonstrated DNA-DSB in A549 and H1299 cells, respectively (Fig. 4H). DMH2 is similar to JL5, having potent inhibition of BMP type 1 receptors with some inhibition of BMPR2 [32]. DMH2 treated cells also demonstrated an increase in DNA-DSB when used in combination with Ym155 (Fig. 4I). To assess whether synergy also occurred by inhibiting TGFβ signaling, we used the selective TGFβ receptor inhibitor SB-505124 [33]. SB-505124 had no effect on DNA-DSB when used alone or in combination with Ym155 (Fig. 4I).

### BMP inhibition combined with YM155 or phenformin induces nuclear localization of AIF

**Fig. 5:**
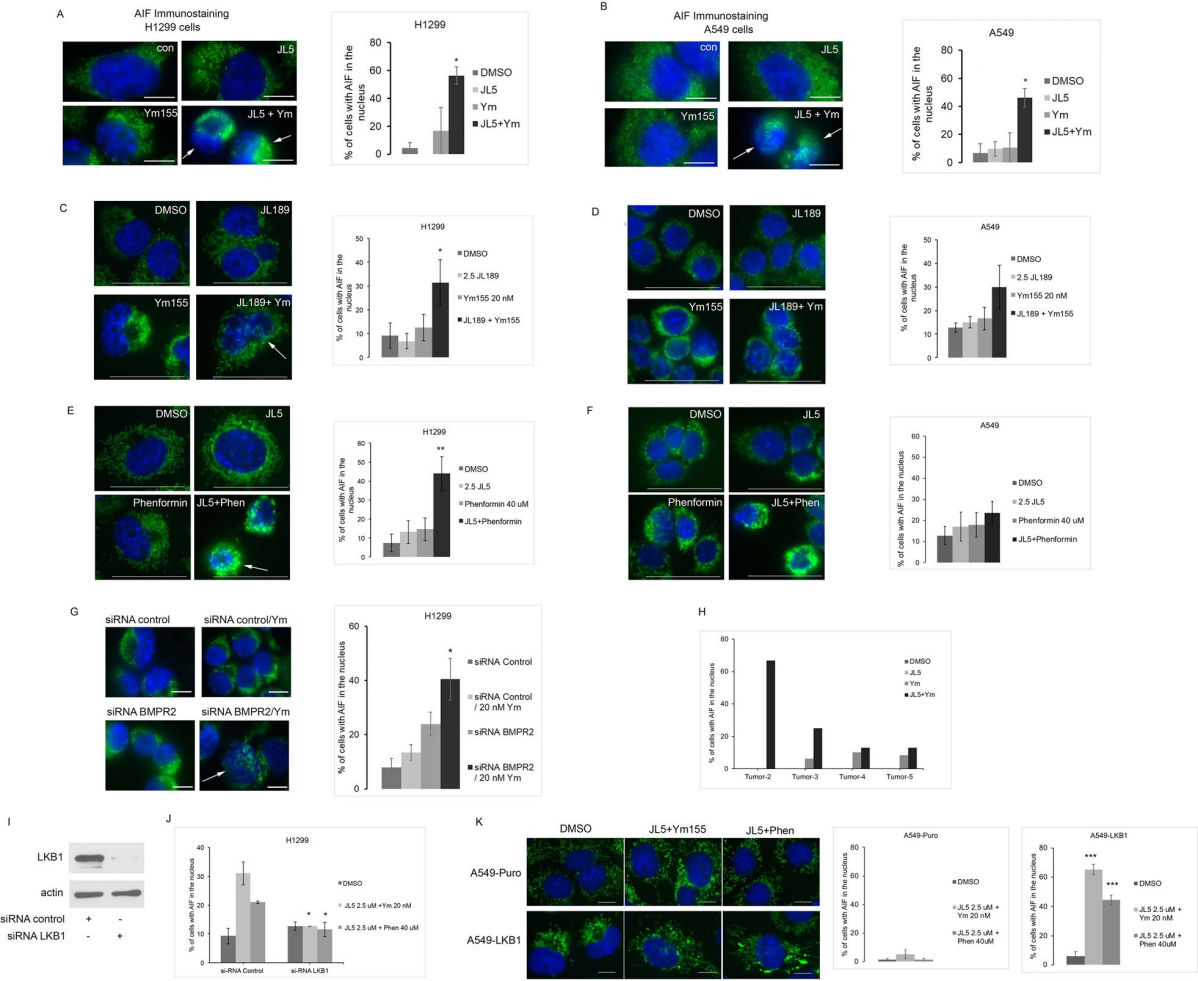
BMP inhibitors together with Ym155 or phenformin induce AMPK/LKB1 dependent nuclear localization of AIF. **A**, **B** AIF immunostaining of H1299 and A549 cells treated with JL5 and Ym155 alone and in combination for 24 h. **C**, **D** AIF immunostaining of H1299 and A549 cells treated with JL189 and Ym155 alone and in combination for 24 h. **E**, **F** AIF immunostaining of H1299 and A549 cells treated with JL5 and phenformin alone and in combination for 24 h. Representative immunofluorescent images of AIF (green) with nucleus stained with DAPI (blue). Arrow depicts cell with nuclear staining for AIF. **G** AIF immunostaining of H1299 cells transfected with siRNA control or siRNA BMPR2 then treated with DMSO or Ym155 20 nM for 24 h. **H** AIF immunostaining of primary lung carcinomas treated with JL5 and Ym155 alone and in combination. Graphs depict representative experiments of each condition, demonstrating percentage of cells with AIF localized to the nucleus. Approximately 50–70 cells from random high-power fields were counted. **I** Western blot of H1299 transfected with siRNA scrambled control and siRNA for LKB1. **J** Localization of nuclear localization of AIF of treated H1299 cells following transfection with siRNA scrambled control and siRNA for LKB1. Data represents mean of 3 experiments. **K** Nuclear localization of AIF in A549-Puro and A549-LKB1 treated for 54 h. Graphs represent the mean of 2 experiments with approximately 100 cells or more counted per condition. Scale bars are equal to 10 μm

Using immunofluorescent imagining, we examined whether AIF is localized to the nucleus following treatment. JL5 and Ym155 in combination caused an increased AIF localization to the nucleus, compared to each compound alone in both H1299 and A549 cells (Fig. 5A, B). The combination of JL189 with Ym155 also caused a significant increase in nuclear localization of AIF compared each compound alone in H1299 cells but not in A549 cells (Fig. 5C, D). The combination of JL5 with phenformin caused a significant increase in the nuclear localization of AIF in H1299 cells but not in A549 cells (Fig. 5E, F). These studies show that pharmaceutical inhibition of BMP signaling together mitochondrial inhibitors synergistically induce nuclear localization of AIF predominantly in LKB1 competent lung cancer cells. The exception is A549 cells treated with JL5 and Ym155, which allosterically enhances AMPK activation.

### AIF nuclear localization if dependent on BMPR2

To assess whether the increased nuclear localization of AIF in cells treated with BMP inhibitors in combination with Ym155 was mediated by the inhibition of BMPR2, we knocked down the expression of BMPR2 with siRNA in H1299 cells treated with Ym155. The knockdown of BMPR2 together with Ym155 significantly enhanced AIF localization to the nucleus in comparison to siRNA control cells treated with Ym155 (Fig. 5G), suggesting that the inhibition of BMPR2 is required for AIF localizing to the nucleus.

### AIF nuclear localization in primary tumors

AIF nuclear localization was examined in primary lung tumors 2–5. AIF nuclear staining was not seen in primary tumors treated with DMSO or JL5 (Figs. 5H). Only a small percentage of cells from the primary lung tumors treated with Ym155 demonstrated nuclear staining with AIF (0–10%). AIF nuclear staining occurred predominantly in primary tumors that were treated with JL5 in combination with Ym155, that also demonstrated increased cell death (tumors 2 and 3, 67% and 25% of cells, respectively) (Fig. 5H).

### AIF nuclear localization is dependent on AMPK

Next, we examined if AMPK/LKB1 regulated nuclear localization of AIF, following BMP inhibition combined with Ym155 or phenformin. Knockdown of LKB1 using siRNA attenuated AIF nuclear localization following treatment with JL5 combined with Ym155 or phenformin in H1299 cells (Fig. 5I-J). To further evaluate if AMPK activation increases nuclear localization of AIF, A549-LKB1 and A549-Puro cells were treated with JL5 with either Ym155 or phenformin. JL5 combined with Ym155 or phenformin significantly increased A549 nuclear localization of AIF in A549-LKB1 cells compared to control and A549-Puro cells (Fig. 5K). These data show that enhanced activation of AMPK/LKB1 signaling induced by BMP inhibition with mitochondrial inhibitors Ym155 and phenformin mediates nuclear localization of AIF.

## Discussion

AMPK is a conserved master regulator of catabolism that conserves ATP utilization by suppressing anabolic metabolism and increasing ATP production by inducing mitochondrial biogenesis [34]. AMPK prevents cell death of cancer cells during cellular stress through its regulation of energy conserving pathways [24, 35]. Several studies report that AMPK acts as a tumor suppressor through its suppression of anabolic metabolism leading to growth inhibition [22, 36]. Hyperactivation or prolonged AMPK activation during cell stress can induce cell death or cellular dysfunction [22, 37]. In cancer cells, AMPK can switch from energy-conserving pro-survival signaling to pro-apoptotic signaling. MYC constitutively drives anabolic metabolism and when combined with prolonged AMPK activation can lead a cell cycle and metabolic crisis [22]. AMPK has been shown to mediate tumor suppression by phosphorylating and activating p53 leading to growth suppression and apoptosis [38]. AMPK is also reported to regulate cell death by inhibiting Bcl-XL and activating the pro-apoptotic Bcl-2 family member BIM [22, 39].

We recently reported in *C elegans* and lung cancer cells that BMP signaling suppresses AMPK activation [20]. Inhibition of BMP signaling with small molecules receptor inhibitors, knockdown of LKB1, or *lof* BMP transgenes led to the activation of AMPK signaling. In the lung cancer cells, BMP suppression of AMPK was mediated by downregulating LKB1 activity [20]. In the present study, we show that enhanced activation of AMPK occurs when BMP inhibitors are combined with the mitochondrial inhibitors Ym155 or Phenformin. The greatest activation of AMPK occurred in cells expressing LKB1, suggesting LKB1 activation is required. Ym155 and Phenformin decrease ATP production leading to the activation of AMPK. Thus, the enhanced AMPK activation occurring with BMP inhibition together with Ym155 or phenformin is likely mediated by both an increase AMP/ATP ratio and LKB1 activation.

Our studies demonstrate that activation of AMPK can promote cell death of lung cancer cells. This was demonstrated with Ym155 inducing more cell death in lung cancer cells expressing LKB1. In addition, activating AMPK directly with A769662 also induced cell death. Furthermore, our studies suggest that synergistic cell death induced by BMP inhibitors and Ym155 or phenformin involves “hyperactivation” of AMPK. JL5 combined with Ym155 did increase cell death in both H1299 and A549 cells. However, JL5 and Ym155 combination did cause an increase in pACC levels in A549 cells. JL5 combined with Ym155 enhanced AMPK activity and increased cell death in A549-LKB1 cells but not in A549-Puro cells consistent with LKB/AMPK promoting cell death. Further supporting the role for AMPK inducing cell death, JL5 combined with phenformin and JL189 combined with Ym155 or phenformin synergistically induced cell death in H1299 but not in A549 cells.

Our studies suggest that nuclear localization of AIF is dependent on AMPK activation. Knockdown of LKB1 prevented AIF nuclear localization of AIF following treatment with JL5/Ym155 and JL5/Phenformin. Other than A549 cells treated with JL5 and Ym155, AIF nuclear localization was increased in lung cell lines expressing LKB1. Interestingly, cell death and AIF nuclear localization, induced by BMP inhibition together with Ym155 or Phenformin occurred independent of caspase activation. AIF is an evolutionary conserved protein that has two independent functions: biogenesis of the electron transport chain and cell death [40–42]. AIF is a phylogenetically old, programed cell death pathway that is independent of caspase activation [42]. An increase in permeability of the outer mitochondrial membrane (OMM) is required for its release into the cytosol [43]. AIF is transported to the nucleus where it induces large-scale DNA fragmentation and cell death [31, 42, 43]. AIF caspase-independent cell death is predominantly reported in ischemic-reperfusion injury in neurons and myocardium, which is mediated by calcium influx. The ability to induce AIF-caspase independent cell death with target specific pharmaceuticals has potential therapeutic benefits as it has been shown to induce cell death in cancer cells resistant to apoptosis [44].

## Conclusion

Our studies suggests that BMP inhibition together with the mitochondrial inhibitors, Ym155 and phenformin, synergistically promote cell death of lung cancer cells that is mediated in part by “hyperactivation” of AMPK, promoting nuclear localization of AIF independent of caspase activation. These studies suggest a novel mechanism by which “hyperactivation” of AMPK promotes AIF caspase-independent cell death in lung cancer cells. Furthermore, these studies provide mechanistic insight into utilizing BMP inhibitors to synergistically induce cell death of lung cancer cells.

## Supplementary Information

**Additional file 1. Figure S1:** Ym155 together with BMP inhibitors JL5 or JL189 synergistically enhances cell death in lung cancer cells. (A, B). Mean percent dead cells of 4 experiments of H1299 and A549 cells treated with BMP inhibitors JL5, JL189 or Ym155 alone and in combination for 48 hrs. Yellow table represents the combination index (CI) of cells treated with Ym155 plus BMP inhibitor at each concentration. Concentration of drugs used increase from left to right.

**Additional file 2.** BMP inhibitors regulation of Alk3 and BMPR2.

**Additional file 3. Figure S2:** Phenformin together with BMP inhibitors JL5 or JL189 synergistically enhances cell death in lung cancer cells. (A, B). Mean percent dead cells of 4 experiments of H1299 and A549 cells treated with BMP inhibitors JL5, JL189 or phenformin alone and in combination for 48 hrs. Yellow table represents the combination index (CI) of cells treated with phenformin plus BMP inhibitor at each concentrations. Concentration of drugs used increase from left to right.

## Data Availability

The datasets obtained and analyzed for this study will be made available from the corresponding author in a reasonable request.

## Abbreviations

BMP: Bone morphogenetic protein
BMPR2: Bone morphogenetic protein receptor 2
AIF: Apoptosis inducing factor
XIAP: X-linked inhibitor of apoptosis protein
TAK1: Transforming growth factor beta (TGFβ) activated kinase 1
NSCLC: Non-small cell lung carcinomas
AMPK: AMP activated kinase
LKB1: Liver kinase B1
ACC: Acetyl-CoA carboxylase
kD: Kilodalton
PI3K: Phosphatidylinositol-3-kinase
c-elegans: *Caenorhabditis elegans*
DNA-DSB: DNA double stranded breaks
PARP: Z-VAD-FMK (VAD) Poly ADP ribose polymerase
RIP1: Receptor-associated adaptor kinase 1
c-IAP: Inhibitor of apoptosis proteins
