# Supplementary figures and images for: Bone morphogenetic protein inhibitors and mitochondria targeting agents synergistically induce apoptosis-inducing factor (AIF) caspase-independent cell death in lung cancer cells
Source: Cell Commun Signal. 2022 Jun 27;20:99. doi: 10.1186/s12964-022-00905-4 (PMC9238106; doi:10.1186/s12964-022-00905-4)

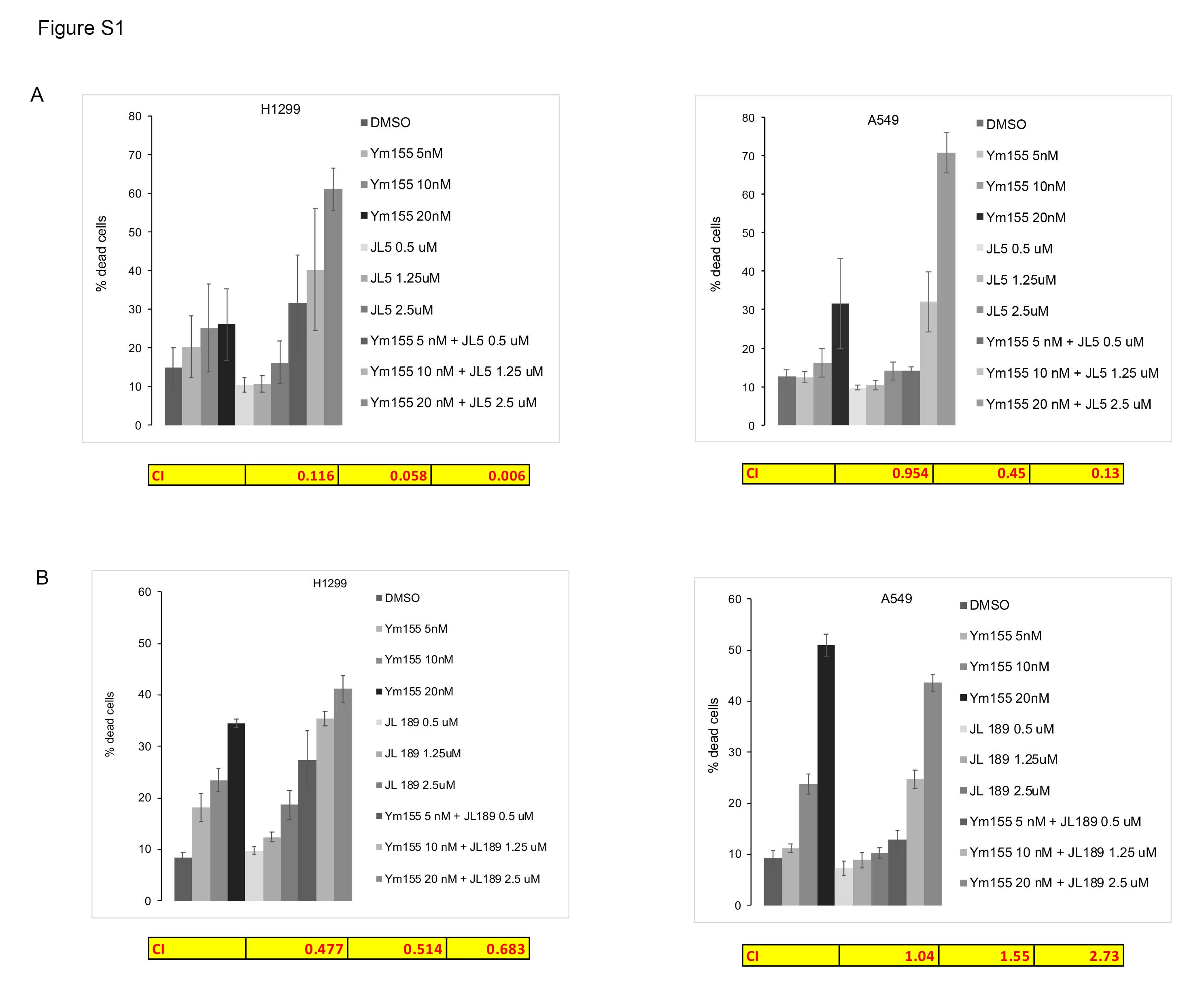

Supplement: Supplementary file 2 — Additional file 1. Figure S1: Ym155 together with BMP inhibitors JL5 or JL189 synergistically enhances cell death in lung cancer cells. (A, B). Mean percent dead cells of 4 experiments of H1299 and A549 cells treated with BMP inhibitors JL5, JL189 or Ym155 alone and in combination for 48 hrs. Yellow table represents the combination index (CI) of cells treated with Ym155 plus BMP inhibitor at each concentration. Concentration of drugs used increase from left to right. [file 12964_2022_905_MOESM2_ESM.jpg]

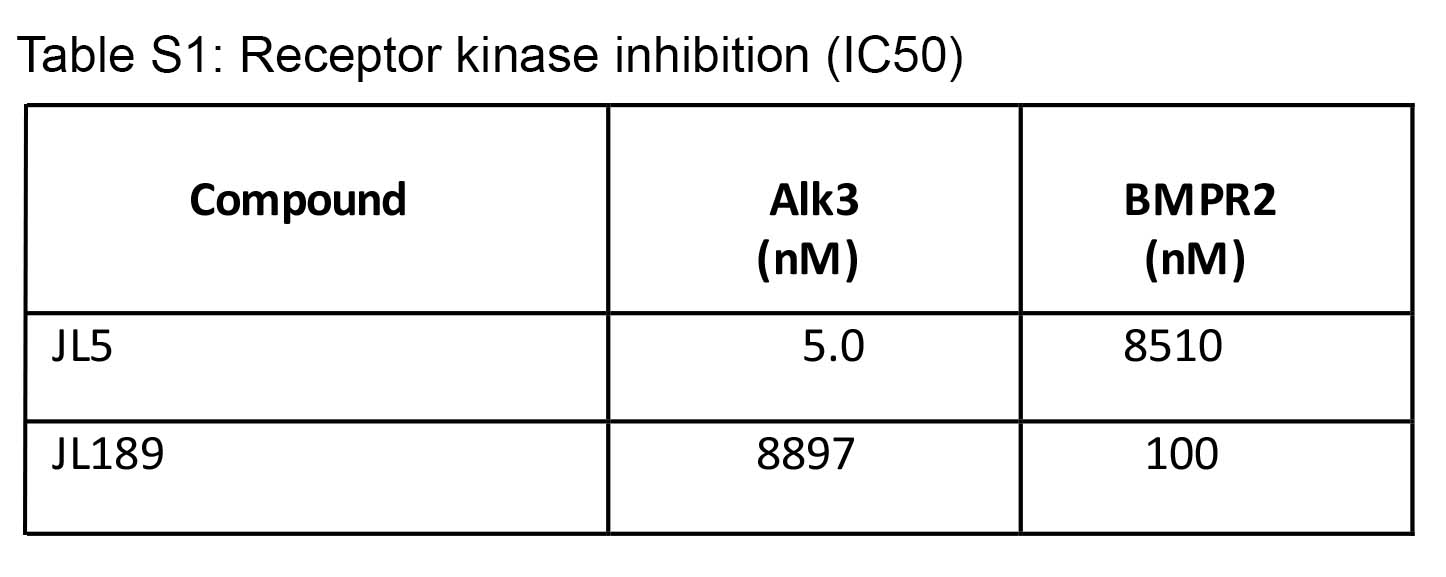

Supplement: Supplementary file 3 — Additional file 2. BMP inhibitors regulation of Alk3 and BMPR2. [file 12964_2022_905_MOESM3_ESM.jpg]

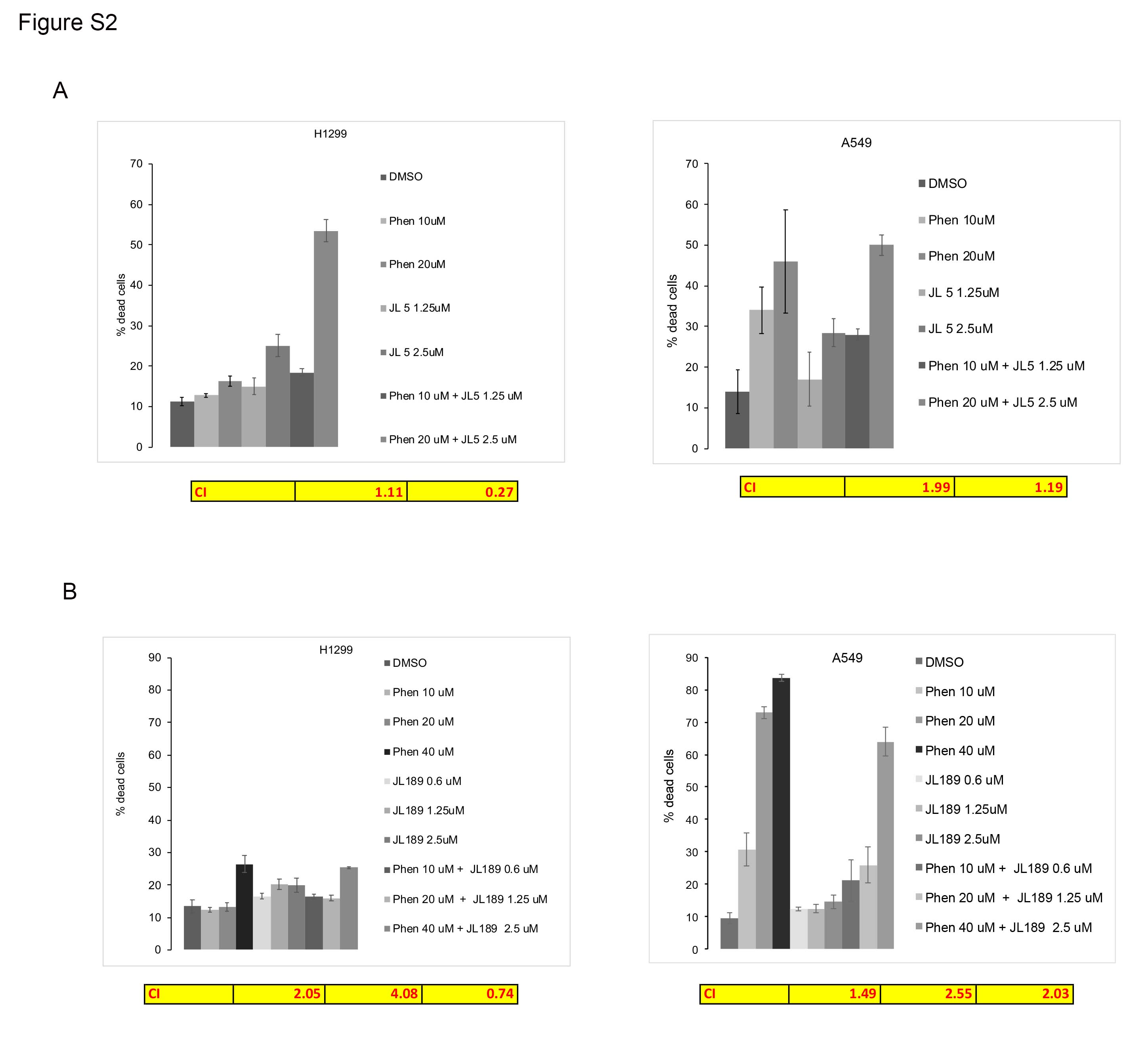

Supplement: Supplementary file 4 — Additional file 3. Figure S2: Phenformin together with BMP inhibitors JL5 or JL189 synergistically enhances cell death in lung cancer cells. (A, B). Mean percent dead cells of 4 experiments of H1299 and A549 cells treated with BMP inhibitors JL5, JL189 or phenformin alone and in combination for 48 hrs. Yellow table represents the combination index (CI) of cells treated with phenformin plus BMP inhibitor at each concentrations. Concentration of drugs used increase from left to right. [file 12964_2022_905_MOESM4_ESM.jpg]
